# Exposure to parental depression in childhood and memory trajectories in mid- and later life

**DOI:** 10.1371/journal.pone.0342573

**Published:** 2026-03-18

**Authors:** Zhenmei Zhang, Seung-won Emily Choi, Yu Guo

**Affiliations:** 1 Department of Sociology, Michigan State University, East Lansing, Michigan, United States of America; 2 Department of Sociology, Anthropology, and Social Work, Texas Tech University, Lubbock, Texas, United States of America; 3 Department of Philosophy, Zhejiang Institute of Administration, Hangzhou, People’s Republic of China; 4 Zhejiang Province “Eight-Eight Strategy” Innovation Development Institute, Zhejiang Institute of Administration, Hangzhou, People’s Republic of China; Methodist University Cape Fear Valley Health School of Medicine, UNITED STATES OF AMERICA

## Abstract

Little is known about whether childhood exposure to parental mental health problems, a significant stressor in early life, influences an individual’s memory trajectories in mid- and later life. Drawing on data from four waves (2011–2018) of the China Health and Retirement Longitudinal Study (CHARLS) and its supplement, the 2014 Life History Survey, we used the latent growth curve modeling technique to investigate whether exposure to parental depression in childhood is associated with memory trajectories of Chinese older adults. Our analytic sample included 7,097 respondents aged 50 and above who were raised by both parents. Results show that individuals who recalled having both parents depressed during their childhood had lower memory scores at baseline compared to their non-exposed counterparts, even after controlling for childhood socioeconomic status and health. These differences remained stable over the 7-year follow-up. Educational attainment and adult health—particularly depressive symptoms—explained the lower episodic memory scores observed in individuals who experienced both parents’ depression during childhood. Surprisingly, individuals who reported having only one parent (either mother or father) depressed during their childhood did not show significantly different memory trajectories compared to their non-exposed counterparts. The findings underscore the long arm of parental mental health on cognitive function in later life and highlight the importance of preventing and treating parental depression through a child’s formative years.

## Introduction

Although the etiologies underlying cognitive health outcomes in later life are complex, the existing literature suggests that upstream social factors, including the material, social, and psychological adversities that adults experience during childhood, can have lasting impacts on cognitive function [1–3]. For example, a substantial body of research shows that children who grow up in poor households, experience hunger or malnutrition, or are exposed to childhood abuse tend to exhibit lower cognitive functioning in later adulthood [4–6]. Furthermore, these effects are particularly pronounced in low- and middle-income countries experiencing rapid population aging [7,8]. Among the literature on adverse childhood conditions and cognitive health across mid- and later life, the vast majority has focused on childhood socioeconomic status (SES), nutrition, and health [6,9,10], with limited attention to psychological stressors in early life [11].

It is estimated that as high as 18% of parents suffered from mental illness in the United States between 2008 and 2014, making exposure to parental mental health problems one of the most common stressors in childhood [12]. Mental illness among parents is more common for mothers than for fathers [12,13]. Previous research has indicated that mothers face an elevated risk of experiencing depressive symptoms postpartum [14]. Recent estimates from China also showed that approximately 19% of mothers and 14% of fathers of Chinese adolescents suffered from depression [15]. A small but growing body of literature suggests that childhood exposure to parental mental illness can have lasting effects on mental health in adulthood through various mechanisms [13,16,17].

In China, the associations between adverse childhood experiences (ACEs) and cognitive function have been well-documented among older adults. However, most studies rely on composite measures of ACEs and do not disentangle the specific effects of parental mental health problems from other forms of childhood adversity [18–20]. One notable exception is a recent study showing that childhood exposure to parental mental health problems predicts poorer cognitive functioning in later life, partly through elevated depressive symptoms in mid- and later adulthood [21].

To extend previous work on the long-term effects of childhood conditions on later-life cognitive outcomes, we drew on data from four waves (2011–2018) of the China Health and Retirement Longitudinal Study (CHARLS) and its supplement, the 2014 Life History Survey, to investigate the link between childhood exposure to parental depression and memory trajectories among middle-aged and older adults in China. CHARLS is particularly well-suited for our study because respondents provided information on both mothers’ and fathers’ mental health issues during their childhood, along with rich information about childhood conditions such as parental education and childhood health, educational attainment, and adulthood health. Although cognition encompasses multiple domains, including memory, executive function, and processing speed [22], our study focuses specifically on episodic memory, a domain that is crucial for independent living and begins to decline in midlife [23,24]. Importantly, memory decline is among the earliest and most clinically relevant markers of cognitive aging and is strongly associated with dementia risk [25,26].

### Parental mental health and cognitive function over the life course

We draw on the life course theory and the stress process model to understand how childhood exposure to parental mental health problems can be associated with cognitive function in later life [11,27,28]. Previous literature has shown that parental mental health problems are major sources of stress for children because parental mental health problems may lead to family dysfunction, relationship stress, and negative parenting [13,29,30]. Moreover, most parental depression is undiagnosed and untreated [31,32]; therefore, it can turn into a chronic stress for offspring. Growing evidence has suggested that stress over the life course can affect cognitive function through biochemical and behavioral mechanisms [33,34]. Stress may lead to the blunting of the hypothalamic-pituitary-adrenal (HPA) axis and synaptic plasticity changes, which may have deleterious effects on memory [35,36].

Childhood is a crucial period for emotional and cognitive growth [13,37]. The life course perspective suggests that parental mental health can have enduring effects on cognitive function in later years through multiple mechanisms. According to the latency model, exposure to early chronic stressors such as parental depression may directly affect the development of multiple brain regions, resulting in reduced dendritic branching and less developed neural connectivity, which in turn may lower cognitive reserve and heighten vulnerability to cognitive decline across the life course [38,39].

There is a well-established body of research demonstrating that maternal mental health can significantly impact a child’s cognitive and psychosocial development [40,41]. When a mother suffers from mental illness, she may have low energy and become withdrawn or irritable, all of which can impair her ability to interact with the child and engage in activities that stimulate a child’s cognitive development, such as playing and reading. For example, studies have shown that maternal depression can negatively affect infants’ neurocognitive development [42–44]. A recent study in Korea found that children whose mothers exhibited significant depressive symptoms had impaired executive function at ages 7–9, compared to those whose mothers were not depressed [45]. Similarly, childhood maternal depression was associated with lower cognitive function among 10–15-year-old adolescents in China [46]. Less research has directly focused on the role of a father’s mental health on a child’s cognitive development. However, father’s depression was significantly associated with lower cognitive ability of adolescents aged between 10 and 15 years old in China after controlling for the socioeconomic status of the parents and the health of adolescents [15]. Previous research has shown that individuals who were exposed to either maternal or paternal poor mental health were more distressed in adulthood but those who were exposed to both maternal and paternal mental health problems had the greatest distress in later life [13]. The impaired development of the brain in childhood can result in a brain that functions less efficiently [38,47].

The pathway model suggests that childhood exposure to parental mental health problems can have an indirect effect on late-life cognition through adulthood socioeconomic status and health. Previous research has established that maternal depression is a significant childhood stressor. It can adversely affect mother-child emotional bonds, relationships, and parenting, which, in turn, can influence offspring’s educational attainment and health outcomes in adulthood. This may be particularly true in China, where educational resources are often limited, and parental support often plays an important role in academic outcomes. For example, retrospective reports of more positive child-parent relationships were significantly associated with higher educational attainment, which, in turn, was linked to better episodic memory in China [3]. Though limited, recent work also suggests that childhood exposure to fathers’ mental health problems may have similar long-term negative effects [13]. Education has long been considered a major determinant of cognitive reserve and is often associated with higher income, more cognitively stimulating occupations and leisure activities, which can provide additional cognitive benefit over the life course [48,49].

In addition, parental mental health problems may significantly increase the risk of adulthood health problems such as depression [13,17], which is associated with lower cognitive functioning and more rapid cognitive decline in later life [50,51]. The connection between parental and offspring depression is multifaceted. Genetic inheritability and the chronic stress of living with a depressed parent both play significant roles. Family and twin studies suggested that the heritability rate of depression may reach up to 40% [52]. Parental mental health issues can also create substantial stress for children, potentially resulting in strained relationships [13,29,30]. Given that many parental mental health issues go undiagnosed and untreated, particularly in developing nations, these challenges and stresses in childhood may persist in the offspring’s young adulthood and beyond, undermining their mental health [53–55]. Parental mental health problems can lead to inadequate attention to children’s nutrition and diet, which have also been associated with poor childhood health [56,57]. However, it is not clear whether poor parental mental health has long-term effects on an individual’s physical health and disability in later life, factors that may increase the risk of cognitive problems [58].

Overall, according to the latency model, parental mental health problems can be associated with poor cognitive outcomes even after controlling for adulthood factors because some negative effects of childhood adversity on cognition may be irreversible [38,59]. According to the pathway model, the association between parental mental health problems and late-life cognitive function should be significantly attenuated when adulthood socioeconomic status and health are controlled for [3,13]. However, these two models are not mutually exclusive, and parental mental health may affect their offspring’s late-life cognition through both processes.

### The present study

Based on previous findings on the health effects of parental mental health problems on children and young adolescents, the present study aims to extend our understanding of the long-term impact of exposure to parental depression on an individual’s memory trajectories in mid- and later life. We hypothesize that:

1) Exposure to parental depression is associated with lower memory scores and a faster memory decline in mid- and later life.
2) Educational attainment and adulthood health could partially account for the association between exposure to parental depression and memory trajectories.

## Materials and methods

### Data

We used four waves (2011–2018) of the China Health and Retirement Longitudinal Study (CHARLS) and its supplement, the 2014 Life History Survey for the analysis. The CHARLS is modeled after the Health and Retirement Study (HRS) in the United States. It is a nationally representative longitudinal survey of community-dwelling adults aged 45 and older in China. Married respondents and their spouses are both interviewed. A multistage cluster sampling method was used to obtain the sampling frame of CHARLS in 2011, and interviews were completed with 17,708 respondents from 150 counties in 28 provinces in China. Face-to-face computer-assisted interviews were conducted, with a response rate of 80.5% at baseline. In 2014, a supplementary Life History Survey collected detailed information about respondents’ childhood circumstances, education, occupational history, and health. Detailed information on CHARLS sampling procedures, recruitment strategy, and follow-up surveys can be found elsewhere (http://charls.pku.edu.cn). About 82% of baseline respondents participated in the 2014 supplementary survey (n = 14,436). Our analytic sample is restricted to participants who were aged 50 and older (3,342 excluded), raised by both parents (i.e., biological, step, or adoptive) during most of their childhood (1,322 excluded), answered questions about parental depression (1,199 excluded), and had valid data in memory tests at baseline (1,457 excluded). We further excluded 19 respondents who had missing data on their parents’ education or childhood rural residence, which led to 7,097 respondents in our study.

### Measures

#### Dependent variable.

***Episodic memory*.** Neuropsychologists commonly use list learning tests to measure episodic memory, as they capture an individual’s ability to encode, store, and retrieve information [60,61]. CHARLS respondents were asked to recall 10 Chinese nouns right after they were read to them (immediate recall) and then four minutes later (delayed recall). The scores on these two measures were significantly correlated (r = 0.69, p<.001). We averaged the immediate and delayed recall scores to create a composite measure of episodic memory [3,62].

#### Independent variables.

Our key independent variable is exposure to parental depression in respondents’ childhood. Respondents were asked whether either parental figure experienced sadness or depression that lasted 2 weeks or more before they were 17 years old. Based on this information, we classified parental depression into four mutually exclusive categories: 1) both mother and father were depressed, 2) only mother was depressed, 3) only father was depressed, and 4) neither parent was depressed (reference group). Although this measure may be subject to recall bias, prior research indicates that children can recognize their parents’ mental health struggles and that neither age nor adult depression significantly compromises the consistency of recalling childhood events. Together, these findings support the validity of retrospective assessments of childhood experiences [63,64].

***Educational attainment*.** Two potential mechanisms (i.e., educational attainment and adulthood health) linking exposure to parental depression and later-life memory are investigated. *Educational attainment* was coded into five categories: no schooling (= reference), less than elementary school, elementary school, middle school, and high school or more.

***Adulthood health*.** We measured adulthood health using *depressive symptoms and activities of daily living disability* (ADL disability) at baseline*. Depressive symptoms* were measured by the 10-item Center for Epidemiologic Studies Depression Scale (CESD-10), which had adequate reliability and validity for older adults in China [65,66]. CHARLS respondents were asked how often they felt depressed, lonely, happy, fearful, and hopeful, as well as how often they had restless sleep, were bothered by things that don’t usually bother them, had trouble keeping their minds on what they were doing, felt everything was an effort, and could not get going during the last week. The sum of the CESD-10 scores ranged from 0 to 30, with higher scores indicating more depressive symptoms (Cronbach’s alpha = 0.80). ADL *disability* was measured as a binary variable (1 = one or more limitations; 0 = no limitations).

#### Other sociodemographic covariates.

We controlled for age at baseline (in years), gender (1 = female; 0 = male), marital status (1 = unmarried; 0 = married), current residence (1 = rural; 0 = urban), mother’s education (1 = one or more years of schooling; 0 = no schooling), father’s education (no schooling, elementary school, and middle school and above, with no schooling as the reference category), childhood residence (1 = rural; 0 = urban), childhood health using a 5-point scale (1 = poor; 5 = excellent), raised by biological father (1 = yes; 0 = no), and raised by biological mother (1 = yes; 0 = no).

### Statistical methods

We estimated latent growth curve models (LGCMs) to examine the association between exposure to parental depression in childhood and later-life memory trajectories among Chinese older adults aged 50 and above from 2011 to 2018. LGCMs estimate the average memory trajectories over time using two latent growth factors: the latent intercept, reflecting the level of cognitive functioning at baseline, and the latent slope, reflecting the rate of change over the follow-up [67]. The model can be specified as follows:


$$Y_{ij}=\pi_{0i}+\pi_{1i}T_{ij}+\varepsilon_{ij}$$ (1)

$$\pi_{0i}=a_{\text{0}}+a_{\text{1}}P_{i}+X\text{'}A_{\text{0}}+\delta_{0i}$$ (2)

$$\pi_{1i}=b_{\text{0}}+b_{\text{1}}P_{i}+X\text{'}A_{\text{1}}+\delta_{1i}$$ (3)

*Y*_*ij*_ is the *i*th individual’s memory function at time *j*. *π*_*0i*_ and *π*_*1i*_ are the latent intercept and slope of the memory trajectory for the *i*th individual across waves. *T*_*ii*_ denotes analysis time, which is the years since the baseline survey. *P*_*i*_ is exposure to parental depression in childhood, ***X*** ′ is the vector of covariates at baseline, *A*_*0*_ and *A*_*1*_ are vectors of corresponding coefficients. *ε*_*ij*,_ *δ*_*0i*_ and *δ*_*1i*_ represent residual terms. The parameters *a*_*1*_ and *b*_*1*_ are the focus of the interpretation, describing the effect of parental depression on the intercept and slope of the memory trajectories.

The analyses estimated three models. The base model (Model 1) included the presence of parental depression, child socioeconomic status and health, and basic controls (i.e., age, gender, marital status, and current residence). Then, educational attainment and adulthood health were added in a series of nested models (Models 2 and 3).

We used STATA to compute the descriptive statistics of the analytic sample, and LGCMs were estimated using Mplus version 7.4 [68]. All models were estimated using Full-Information Maximum Likelihood (FIML) to account for missing data and attrition. Additional analysis showed that 4,278 respondents (60%) had complete memory data in four waves. Those who had incomplete memory data in the follow-up were more likely to be older, unmarried, less educated, disabled, have grown up in and lived in urban areas, and were less likely to report maternal-only depression in childhood.

## Results

### Descriptive results

Table 1 presents summary statistics of the analytic variables. The mean of the episodic memory score was 3.58 words out of 10 at baseline. About 9.1% of respondents reported that both parents were depressed in their childhood, 9.2% indicated that only their mothers were depressed, and 1.7% stated that only their fathers were depressed. Altogether, about 20% of the respondents were exposed to parental depression in their childhood. Regarding sociodemographic characteristics, the mean age of respondents at baseline (2011) was about 61 years old, and 50.8% were female. Approximately 14% were unmarried. A slight majority lived in rural areas (55%). With respect to adulthood conditions, educational attainment was relatively low: About 25.9% of the respondents had no formal schooling, 19.3% attended but did not complete elementary school, 21.8% finished elementary school, 19.2% received a middle school education, and only about 13.8% completed high school education or above. The mean score of depressive symptoms was 8, and about 14.2% had one or more ADL disabilities.

**Table 1:** Weighted descriptive statistics of Chinese adults aged 50 and older, CHARLS, 2011.

Variables	Mean or Percent
**Cognitive function**	
Episodic memory 2011 (0–10)	3.58 (0.05)
**Parental depression in childhood (%)**	
Mother only	9.23
Father only	1.68
Both mother and father	9.11
None	79.98
**Adulthood conditions**	
Educational attainment (%)	
No schooling	25.86
Less than elementary school	19.30
Elementary school	21.84
Middle school	19.19
High school or more	13.80
Depressive symptoms (0–30)	8.07 (0.14)
ADL disability (%)	14.23
**Controls**	
Age (years)	61.01 (0.16)
Female (%)	50.76
Unmarried (%)	14.06
Current rural residence (%)	54.92
Childhood health (1–5)	3.27(0.03)
Mother had 1 + years of schooling (%)	9.77
Father’s education status (%)	
No schooling	58.77
Elementary school	34.05
Middle school or more	7.18
Grew up in rural area (%)	86.64
Raised by biological mother (%)	97.74
Raised by biological father (%)	96.91

Note: Values in parentheses are standard deviations. *N* = 7,097.

As for early-life conditions, parental education levels were very low, and the majority of the respondents reported that their parents did not receive much education: About 34% of respondents had fathers who had some elementary education, and only 7.2% had middle school education or above, while about 9.8% had mothers who had some schooling. The majority of respondents grew up in rural areas (86.6%). Most were raised by biological parents (i.e., 97.7% by biological mother and 96.9% by biological father).

### Associations between parental depression and memory trajectories

Table 2 shows the coefficients from a series of latent growth curve models. Model 1 is our base model that includes childhood exposure to parental depression, childhood socioeconomic status and health, and sociodemographic control variables, including age, gender, marital status, and rural residence at baseline. We found that having two depressed parents in childhood was associated with lower memory scores at baseline (β = −0.19, p < 0.01) compared to their non-exposed counterparts, net of controls. Surprisingly, having only one depressed parent, regardless of the gender of the parent, was not associated with baseline memory scores. In other words, those individuals who recalled having only one parent depressed in childhood were not significantly different from their non-exposed counterparts in baseline memory. Furthermore, parental depression in childhood was not linked to the rate of memory decline. These results suggest that respondents who reported having both a mother and a father depressed in childhood had significantly lower baseline memory functioning, and this disadvantage persisted over time.

**Table 2:** Latent growth curve models on exposure to parental depression in childhood and episodic memory trajectories, CHARLS, 2011-2018.

Variable	Model 1		Model 2		Model 3	
	β	SE	β	SE	β	SE
**Intercept**						
Parental depression in childhood (ref = none)						
Mother only	−0.03	0.06	0.02	0.06	0.09	0.06
Father only	0.09	0.14	0.10	0.14	0.15	0.14
Both mother and father	−0.19**	0.06	−0.11*	0.05	−0.02	0.05
Childhood health	0.04*	0.02	0.04*	0.02	0.02	0.02
Father’s education (ref = no schooling)						
Elementary	0.20**	0.04	0.09*	0.04	0.07*	0.04
Middle school	0.29**	0.07	0.17*	0.07	0.15*	0.07
Mother had 1 + years schooling (ref = no schooling)	0.10	0.06	0.01	0.06	0.00	0.06
Grew up in rural area (ref = urban)	−0.69**	0.06	−0.41**	0.06	−0.41**	0.06
Raised by biological mother (ref = no)	0.08	0.12	0.08	0.11	0.06	0.11
Raised by biological father (ref = no)	0.08	0.10	−0.01	0.10	−0.01	0.10
Education attainment (ref = no schooling)						
Less than elementary			0.32**	0.05	0.32**	0.05
Elementary			0.59**	0.05	0.55*	0.05
Middle school			0.95**	0.06	0.90**	0.05
High school or more			1.26**	0.06	1.17**	0.06
Depressive symptoms					−0.03**	0.00
ADL disability (ref = no)					−0.12*	0.05
Age	−0.04**	0.00	−0.03**	0.00	−0.03**	0.00
Female	−0.16**	0.03	0.13**	0.04	0.19**	0.04
Unmarried	−0.08	0.05	−0.05	0.05	0.00	0.05
Rural residence	−0.22**	0.04	−0.09*	0.04	−0.05	0.04
**Slope**						
Parental depression in childhood (ref = none)						
Mother only	0.00	0.01	0.01	0.01	0.00	0.01
Father only	0.02	0.03	0.02	0.03	0.01	0.03
Both mother and father	−0.01	0.01	0.00	0.01	−0.01	0.01
Childhood health	−0.00	0.00	−0.00	0.00	0.00	0.00
Father’s education (ref = no schooling)						
Elementary	0.04**	0.01	0.02*	0.01	0.02*	0.01
Middle school	0.04*	0.02	0.02	0.02	0.02	0.02
Mother had 1 + years schooling (ref = no schooling)	0.02+	0.01	0.01	0.01	0.02	0.01
Grew up in rural area (ref = urban)	−0.03*	0.01	0.01	0.01	0.01	0.01
Raised by biological mother (ref = no)	−0.02	0.03	−0.02	0.03	−0.02	0.03
Raised by biological father (ref = no)	0.04+	0.02	0.03	0.02	0.03	0.02
Education (ref = no schooling)						
Less than elementary			0.10**	0.01	0.10**	0.01
Elementary			0.15**	0.01	0.16**	0.01
Middle school			0.17**	0.01	0.17**	0.01
High school or more			0.19**	0.01	0.19**	0.01
Depressive symptoms					0.00**	0.00
ADL disability (ref = no)					0.02	0.01
Age	−0.01**	0.00	−0.00**	0.00	−0.00**	0.00
Female	−0.03**	0.01	0.02**	0.01	0.02*	0.01
Unmarried	−0.03*	0.01	−0.03*	0.01	−0.03*	0.01
Rural residence	−0.05**	0.01	−0.03**	0.01	−0.03**	0.01
RMESA	0.02		0.03		0.03	
BIC	91037		89613		89459	

Note: Unstandardized coefficients were presented.

+p < 0.10; *p < 0.05; **p < 0.01.

To understand the magnitude of the association between exposure to parental depression and episodic memory, we compared the observed estimate (β = −0.19, p < 0.01) to the baseline age effect in Model 1(β = −0.04, p < 0.01). The effect of exposure to depression in both parents was therefore equivalent to approximately 5 years of age difference in memory scores.

In Model 2, we added educational attainment, and the coefficient of having both parents depressed on baseline memory was attenuated but remained statistically significant (β = −0.11, p < 0.05). This suggests that educational attainment partially accounted for the negative effect of having two depressed parents on the baseline memory scores.

In Model 3, we added baseline depressive symptoms and ADL disability to Model 2. The coefficient of having both parents depressed on baseline memory was further reduced and became statistically nonsignificant (β = −0.02, p > 0.05). In additional analysis, we added the two health indicators separately (results available upon request) and the results showed that it was adulthood depressive symptoms that mainly accounted for the effect of having two depressed parents on baseline memories, net of education attainment and all the controls.

Taken together, these results showed that lower educational attainment and adulthood poor health accounted for the negative impact of having both parents depressed in childhood on baseline memory, net of controls.

Several mechanisms may explain why exposure to depression in both parents was associated with baseline memory but not with the rate of decline. Such exposure may shape the peak level of memory function typically attained in early adulthood (around ages 25–35) [69,70], primarily through its effects on early cognitive development and educational attainment. However, it may not affect the major factors in later adulthood—such as cardiovascular disease, unhealthy behaviors (e.g., smoking, physical inactivity), or social isolation—that contribute to memory decline in later life. Alternatively, the absence of an association with memory decline could reflect our relatively short follow-up period, which may have limited our ability to detect subtle group differences in the rate of memory decline.

## Discussion

Our study is one of the first to examine the associations between childhood exposure to parental depression, a notable childhood stressor, and memory trajectories in later life. Although previous studies have demonstrated that parental mental health in childhood can affect offspring’s mental health in adulthood [13], as far as we know, no study has yet explored how early-life exposure to parental depression influences cognitive health in later life or the pathways involved. Using data from the 2011–2018 CHARLS, we conducted latent growth curve analysis to examine whether childhood exposure to parental depression affects the memory scores at baseline and the rate of memory change over time in mid- and later life. We also examined whether adulthood conditions, including educational attainment and adulthood health, accounted for the associations.

Our study has extended the previous studies in several ways. First, our results show that exposure to parental depression can have a long-term negative impact on episodic memory in mid- and later life, even after accounting for childhood confounders like socioeconomic status and health. Specifically, having two depressed parents in childhood was significantly associated with the intercept (i.e., baseline memory) but not the slope of memory functioning (i.e., the rate of decline) compared to their non-exposed counterparts among Chinese older adults. However, having only one depressed parent was not associated with either the intercept or the slope of the episodic memory. Therefore, our first hypothesis, which suggests the links between exposure to parental depression and lower memory scores and faster memory decline, is partially supported. Our findings are largely consistent with previous cross-sectional studies which found that parental depression was associated with lower cognitive functioning among children and adolescents [15,45,46,71]. Furthermore, this study demonstrates that the negative effect of early-life exposure to parental depression on cognition may well extend to later life, but only for those who recalled having both parents depressed. Our findings underscore that it is important to consider both maternal and paternal depression when we conceptualize childhood disadvantages. Most previous studies have focused on maternal depression and have consistently shown that it is associated with children’s cognitive development [43,45,46]. The emphasis on mothers is understandable, given that they experience higher rates of depression than fathers and, in many cultural contexts, including China, often serve as the primary caregivers. Consequently, maternal depression may directly influence daily parenting, emotional support, and cognitive stimulation in childhood. Therefore, our finding that maternal depression alone is not associated with episodic memory is surprising. We speculate that when fathers are not experiencing depression, their active involvement in the family can provide children with an alternative source of emotional support, potentially forming an “emotional alliance” that buffers the adverse effects of maternal depression [30,72,73].

Second, our results show that educational attainment partially explains the association between early-life exposure to both parents’ depression and baseline episodic memory. This supports our second hypothesis, which expects that educational attainment and adulthood health partially account for the relationship between exposure to parental depression and memory trajectories. Our finding is largely consistent with the pathway perspective [3,13], suggesting that having both parents depressed in childhood may lead to lower educational achievement, which in turn negatively affects an individual’s episodic memory in later life. Lower educational attainment may influence memory trajectories through mechanisms such as less intellectually demanding work, fewer opportunities for language development and conversation, and limited access to physical and mentally engaging leisure activities that support cognitive functioning [74].

Third, we find that adult health, depressive symptoms in particular, partially contributes to the association between early-life exposure to both parents’ depression and baseline episodic memory, after controlling for educational attainment, other childhood disadvantages, and sociodemographic controls. This finding aligns with extensive research on the connection between parental and offspring depression [13,17], as well as the effects of depression on cognitive functioning [50,51]. Exposure to parental depression during childhood is associated with long-term mental health challenges, including an elevated risk of depression in adulthood [13,17]. These associations likely reflect both genetic vulnerability and the chronic stress of growing up with a depressed parent. In turn, a history of depression in an individual may adversely affect cognitive functioning in later life.

Last, although we did not find strong support for the latency model, which suggests that exposure to parental depression may directly affect cognitive function, we could not rule it out because the two models — the latency model and the pathway model — are not mutually exclusive, and both processes may operate simultaneously. For example, we did not have data on respondents’ early-life cognition (i.e., an indicator of brain development), which can be affected by parental depression [42]. Early-life cognition is strongly linked to cognition in later life and can also affect educational achievement [75], which serves as a pathway to later-life cognition.

Our study has a few limitations. One primary limitation of this study is its reliance on respondents’ retrospective recall of childhood experiences, which is subject to recall errors. For example, some respondents may not accurately recall their parents’ depression, particularly if these episodes occurred early in childhood [13]. In addition, prior research suggests that individuals are more likely to underreport rather than overreport adverse childhood events, suggesting that our estimates may be conservative [76]. Importantly, retrospective reports are not static: Over the life course, people often acquire additional information about their childhood circumstances from their parents, grandparents, siblings, and other family members. These later-life disclosures may shape how respondents reconstruct and make meaning of their childhood experiences, potentially introducing both random and systematic bias into recall [77]. Future studies should use prospective data to examine the long-term effects of parental mental health problems, considering their timing, severity, and duration, on their children’s cognitive trajectories over the life course.

Second, our sample of older adults represents relatively ‘healthy survivors’ because we excluded those who did not participate in memory tests in 2011, as well as those who did not participate in the supplementary survey in 2014. Thus, those who were too ill or incapable of answering the memory questions were likely excluded from our analytic sample. These exclusions may lead to an underestimation of the effects of exposure to parental depression on episodic memory, as previous studies have found that those who had parental mental problems in childhood were more likely to have poor health in adulthood and beyond [13,78].

Third, our data, CHARLS lacks information on the respondents’ mental health history during young adulthood, which would be a more powerful mediator between childhood exposure to parental depression and episodic memory in later life. Previous research has indicated that children whose parents have poor mental health have elevated distress throughout their adulthood [13], and a history of mental health problems predicts lower levels of cognitive functioning [79]. We suggest that future research consider the role of mental health issues over the life course to better understand how early exposure to parental depression impacts cognition in mid- and later life. Fourth, our sample size of paternal-only depression is very small, and the results should be interpreted with caution. Last, it is important to note that the present study was not pre-registered, and therefore, the analyses should be considered exploratory.

Despite these limitations, our study extends previous literature on the long-term impacts of parental mental health on their children’s health by focusing on episodic memory in later life. Our results show that exposure to parental depression is a significant childhood stressor for the current cohort of middle-aged and older adults in China. Those individuals who recalled having both parents depressed in their childhood had lower levels of episodic memories compared to their non-exposed counterparts, and this gap persisted over time. Intervention in preventing and treating parental depression can have positive and ripple effects on their children’s cognitive function over the life course.
